# The Treatment of Wounds in War

**Published:** 1915-07-10

**Authors:** 


### THE TREATMENT OF WOUNDS IN WAR.

The concluding Chadwick lecture* of the present session was delivered by Mr. H. S. Souttar, F.R.C.S. (late surgeon-in-chief of the Belgian Field Hospital), at the house of the Royal Society of Medicine.

The lecturer disclaimed any intention of placing before his audience any complete scheme for the treatment of wounds in war: such an aim would not only require many lectures, but the attempt would be presumptuous. His purpose was to give a general impression of the conditions under which these wounds had to be treated, and the attempts which had been made to solve the great problems to which these gave rise.

The present position of the surgery of wounds was due chiefly to two men: Baron Larrey and Lister, of whom the former was the less known, yet great authorities had declared him to be one of the greatest surgeons who ever lived. He was surgeon-in-chief to the French Army, in all the campaigns of the great Napoleon, though his work reached its climax in the Russian campaign of 1812-13. His personal services were devoted to the Imperial Guard, for whom he organised flying ambulances. After the battle of Borodino he was said to have performed 200 amputations with his own hands in twenty-four hours; and nine out of eleven cases of amputation at the shoulder joint were reported to have made good recoveries. His life was one long story of indomitable energy, dauntless courage, and incomparable skill. Napoleon stated that Larrey was the greatest man he had ever known. He said that if amputation of a limb was likely to be necessary it should be carried out in the first twenty-four hours, for then the operation was done on what was practically a clean limb. He also insisted on operations being performed in the absolute minimum of time. In those pre-anæsthetic days it was impossible to exaggerate what brilliant technique meant to the patient. He rarely took more than four minutes to amputate at the shoulder or the hip, including every detail; the actual removal occupying only some fifteen seconds.

The great work of Lord Lister was to enable operations to be done in the absence of bacteria; and it was that work which rendered modern surgery possible. The problem of the sepsis of wounds could not be solved, he thought, by merely putting disinfectants into the wound; but if secondary infection of a wound were obviated, as much was thereby done for the patient as if the bacteria in his wound had been destroyed. It was upon these lines that a great advance was to be expected in the treatment of these difficult cases, because nothing could prevent these war wounds from being septic. When a man was brought in wounded, the first thing to deal with was not the wound, but the man himself; he must be restored to a condition in which he was treatable. After the period of strain to which he had been subjected, even a slight wound might mean a nervous breakdown; and if the wound were serious he was suffering from blood loss and shock. The severe pain must first be stopped, because while that lasted practically anything which might be done was useless. At the Front three things were done in the case of every wounded man: he was given morphia and plenty of fluid, and was made warm, the second of these in order to compensate for the blood loss. Often half-an-hour after a man was brought in apparently moribund he would be sitting up and smoking a cigarette. He agreed with those who said that every wound in this present war was septic. The fact that a wound was septic at first made no difference to the care which should be taken with the dressing of it; the first dressing could not be satisfactorily applied by any but an expert dresser, though it only meant having very clean hands, and refraining from interfering with the wound. Pushing anything into the wound he regarded as an absolute mistake. His own idea was to disinfect the surrounding skin, and put on some simple dressing which contained no organisms. In the case of many of these wounds, though the entrance was small, and the exit not large, there was widespread destruction of intervening tissue; and this pulpy material formed an excellent nidus for the growth of organisms. When bullets or pieces of shrapnel had been driven in, it was better to leave them there unless there were special reasons for interference. In some German bullets the centre of gravity was so far back that on striking the skin the bullet tended to turn over, so that the effect in the tissues was "mushrooming." Wounds of the chest generally did extraordinarily well if nothing was done to them. These patients were usually practically out of danger in twenty-four hours if they were simply kept quiet. But it was a very different matter with abdominal wounds. The results were very bad, and there was considerable difference of opinion as to what should be done. Sometimes the range at which the men were shot was less than 100 yards, so that the missile had a very high velocity on impact. To operate with success on abdominal cases it became necessary to have at disposal near the firing-line a really first-class hospital and nursing staff; and the operations demanded might occupy ninety minutes. With such facilities, perhaps one in three or one in five might recover; and with such first-class hospitals and staff he was satisfied that immediate operation was the treatment. Unless those conditions could be satisfied, operation on abdominal cases was a mistake. It was a terrible thing to have to weigh in the balance a man's life when the surgeon thought that by a great effort he could save it. Failing operation, the only thing to be done was to give large doses of morphia, and keep the patient quiet. Under such conditions some recovered.

The lecture concluded by a discussion of the treatment of various forms of compound fracture, and the points were illustrated by skiagrams of cases. Photographs again formed an important part of the address.

* The two preceding lectures of Mr. Souttar's course were reported in The Hospital of June 26 and July 3.

